# Supplementary material for: Seasonal changes in proportion of cardiac surgeries associated with diabetes, smoking and elderly age
Source: PLoS One. 2022 Sep 22;17(9):e0274105. doi: 10.1371/journal.pone.0274105 (PMC9498963; doi:10.1371/journal.pone.0274105)
Supplement: S1 File — (DOCX) [file pone.0274105.s003.docx]

SEASONAL CHANGES IN PROPORTION OF CARDIAC SURGERIES ASOCIATED WITH DIABETES, SMOKING AND ELDERLY AGE

**Online Supplementary Data**

^1^Ferenc Peták PhD, DSc, ^1,2^Kovács N. Barbara, ^2,3^Szilvia Agócs MD, ^1^Katalin Virág MSc, ^1^Tibor Nyári PhD, DSc, ^1,2^Andrea Molnár, ^1,2^Roberta Südy MD, ^4^Csaba Lengyel MD, PhD,
^2,3^Barna Babik MD, PhD

^1^ Department of Medical Physics and Informatics, University of Szeged, Szeged, Hungary

^2^ Department of Anesthesiology and Intensive Therapy, University of Szeged, Szeged,

Hungary

^3^ Cardiac Surgery Unit, Department of Internal Medicine and Cardiology Center, University of Szeged, Szeged, Hungary

^4^ Department of Internal Medicine, University of Szeged, Szeged, Hungary

|  | **T2DM alone** | **SM alone** | **Elderly alone** | **T2DM + SM** | **T2DM + Elderly** | **SM + Elderly** | **All** | **None** |
| --- | --- | --- | --- | --- | --- | --- | --- | --- |
| N | 2071 | 1246 | 1308 | 836 | 710 | 204 | 159 | 3303 |
| Gender (F/M) | 1005/1066 | 327/918^*^ | 322/986^*^ | 228/608^*^ | 144/566^*^ | 14/190^*^ | 12/147^*^ | 1572/1731 |
| Weight (kg) | 84.4 ± 17^*^ | 77.5 ± 17 | 75.5 ± 13^*^ | 87.0 ± 18^*^ | 81.4 ± 14^*^ | 75.6 ± 13.3 | 81.8 ± 15.0^*^ | 77.6 ± 16.3 |
| Height (cm) | 165 ± 10 | 168 ± 9 | 165 ± 9 | 168 ± 9 | 167 ± 9 | 169 ± 8 | 169 ± 8 | 166 ± 10 |
| BMI (kg/m^2^) | 31.0 ± 5.5^*^ | 27.3 ± 5.2^*^ | 27.6 ± 4.3 | 30.8 ± 5.7^*^ | 29.3 ± 4.4^*^ | 26.6 ± 4.0^*^ | 28.8 ± 4.8 | 28.1 ± 5.0 |
| Age (years) | 65.5 ± 7.6 | 59.0 ± 9.3 | 78.1 ± 4.0 | 61.5 ± 7.5 | 77.3 ± 4.1 | 76.9 ± 4.1 | 76.6 ± 4.0 | 61.7 ± 12.5 |
| HbA1c (%) | 6.97 ± 1.26^*^ | 5.85 ± 0.48 | 5.84 ± 0.68 | 7.16 ± 1.65^*^ | 6.62 ± 1.0^*^ | 5.92 ± 0.45 | 7.28 ± 1.17^*^ | 5.63 ± 0.39 |
| Hb (mg/dL) | 12.29 ± 1.72^*^ | 13.08 ± 1.70^*^ | 12.32 ± 1.75 | 12.78 ± 1.80 | 12.19 ± 1.73^*^ | 12.43 ± 1.82 | 12.09 ± 1.91 | 12.58 ± 1.64 |
| AS/AI/AS&AI (n) (%) | 373/90/43  (15.7/4.3/2.1) | 165/41/15  (13.1/3.3/1.2) | 349/50/27  (25.9/3.8/2.1) | 93/16/9  (10.9/1.9/1.1) | 146/29/13  (20.6/4.1/1.8) | 43/2/2  (21.1/1/1) | 24/7/2  (15.1/4.4/1.3) | 712/184/78  (21.2/5.6/0.2) |
| MS/MI/MS&MI (n) (%) | 19/139/7  (0.9/6.6/0.3) | 10/90/4  (0.8/7.2/0.3) | 3/80/9  (0.2/6.1/0.7) | 2/46/2  (0.2/5.4/0.2) | 6/27/0  (0.8/3.8/0) | 0/10/1  (0/4.9/0.5) | 1/7/0  (0.6/4.4/0) | 32/401/21  (1.0/12.1/0.6) |
| AS&MI (n) (%) | 30 (1.4) | 10 (0.8) | 20 (1.5) | 5 (0.6) | 6 (0.8) | 3 (1.5) | 1 (0.6) | 36 (1.1) |
| AD/AAA  (n) (%) | 12/26 (0.6/1.3) | 15/24  (1.2/1.9) | 3/9^*^  (0.2/0.7) | 21/2^*^  (2.5/0.2) | 1/7  (0.1/1) | 4/3  (2/1.5) | 2/1  (1.3/0.6) | 49/72  (1.5/2.2) |
| LAM (n) (%) | 12 (0.6) | 9 (0.7) | 6 (0.5) | 1 (0.1) | 1 (0.1) | 0 (0) | 0 (0) | 35 (1.1) |
| EI (n), (%) | 60 (2.9) | 33 (2.6) | 29 (2.2) | 21 (2.5) | 16 (2.3) | 3 (1.5) | 6 (3.8) | 105 (3.2) |
| GUCH (n), (%) | 10^*^ (0.5) | 21^*^ (1.7) | 4^*^ (0.3) | 1^*^ (0.1) | 0^*^ (0) | 0 (0) | 0 (0) | 143 (4.3) |
| CAD (n), (%) | 1016^*^ (49.1) | 721^*^ (57.9) | 503 (38.5) | 522^*^ (62.4) | 325^*^ (45.8) | 102^*^ (50) | 85^*^ (53.5) | 1178 (35.7) |
| CAD+MI (n), (%) | 73 (3.5) | 40 (3.2) | 49 (3.7) | 37 (4.4) | 16 (2.3) | 5 (2.5) | 4 (2.5) | 91 (2.8) |
| CAD+AS (n), (%) | 266^*^ (12.8) | 71 (5.7) | 213^*^ (16.3) | 69 (8.3) | 147^*^ (20.7) | 29^*^ (14.2) | 26^*^ (16.4) | 238 (7.2) |

**Table S1.** Characteristics and diagnoses of cardiac surgery patients with diabetes mellitus only (T2DM alone), smoking (SM alone), and aging (Elderly alone). Groups containing pairwise (T2DM + SM, T2DM + Elderly, and SM + Elderly) and concomitant combination (“All”) significant factors were also examined. “None” denotes no occurrence of these risk factors. Data for continuous variables are shown as mean ± SD; data for categorical variables are represented as number of patients in each group (top number in each cell) with percentage relative proportion rates (bottom numbers in each cell). * p < 0.05 vs. “None”. AAA, aortic arch reconstruction; AD, aorta dissection; AI, aortic insufficiency; AS, aortic stenosis; BMI, body mass index; CAD, coronary artery disease; EI, endocarditis; GUCH, Grown-Up Congenital Heart Disease; Hb, hemoglobin; LAM, left atrial myxoma; MI, mitral insufficiency; MS, mitral stenosis; SM, smoking.

**Figure S1.** Seasonal variations in the arterial blood pressure (ABP), serum triglyceride, total cholesterol and serum glucose levels in patients with type 2 diabetes mellitus (T2DM only, red)), smoking (SM only, black), and aging (Elderly only, blue) for the monthly averaged data over the 12-year study period (January 1, 2007 to December 31, 2018). Grey symbols: non-elderly control patients scheduled for cardiac surgery without diabetes and smoking. Solid lined: statistically significant seasonality (p<0.05), dotted lines: no statical significant seasonal change.

|  | **Number of cardiac surgery patients** | **AS/AI/**  **AS&AI/**  **AS&MI** | **MS/MI/**  **MS&MI** | **AD/**  **AAA** | **CAD,**  **CAD+MI,**  **CAD+AS** | **LAM,**  **EI,**  **GUCH** |
| --- | --- | --- | --- | --- | --- | --- |
| January | 970 | 16.8% | 5.2% | 43.2% | 32.9% | 2.0% |
| February | 857 | 16.5% | 7.0% | 42.9% | 32.0% | 1.7% |
| March | 878 | 18.3% | 5.7% | 43.2% | 30.9% | 2.1% |
| April | 911 | 18.3% | 6.1% | 42.2% | 31.1% | 2.5% |
| May | 893 | 17.8% | 6.9% | 42.1% | 31.5% | 1.8% |
| June | 921 | 17.4% | 7.0% | 43.0% | 30.3% | 2.4% |
| July | 644 | 17.9% | 5.0% | 43.4% | 31.5% | 2.3% |
| August | 509 | 18.0% | 4.1% | 43.1% | 32.4% | 2.5% |
| September | 896 | 19.3% | 5.6% | 43.0% | 29.4% | 2.8% |
| October | 890 | 18.7% | 6.4% | 41.8% | 31.1% | 2.1% |
| November | 840 | 18.0% | 6.6% | 42.7% | 30.2% | 2.6% |
| December | 628 | 16.8% | 5.9% | 43.3% | 32.0% | 2.1% |
| **Total** | **9837** | **17.8%** | **6.0%** | **42.8%** | **31.2%** | **2.2%** |

**Table S2.** Total number of cardiac surgery patients included in the data analyses (grey column), and relative number of cardiac surgery patients with different diagnoses (last 5 columns). Data show no significant seasonality; only the holyday seasons show a decrease in the number of patients due the limited availability of human resources available at the university hospital. No significant difference was observed between the months in the relative frequency of different diagnoses.
