# Supplementary material for: Seasonal changes in proportion of cardiac surgeries associated with diabetes, smoking and elderly age
Source: PLoS One. 2022 Sep 22;17(9):e0274105. doi: 10.1371/journal.pone.0274105 (PMC9498963; doi:10.1371/journal.pone.0274105)
Supplement: Attachment — Submitted filename: PLOS_2nd_Reviewers_QA.docx [file pone.0274105.s005.docx]

**Reviewer #2:**

“The authors have greatly improved the manuscript. However an important point in my opinion was not addressed. I appreciate the fact that only new surgeries were taken into account was highlighted and the sensitivity analysis using different base populations, with and w/o the risk factors, but I believe that what the authors describe is not incidence. The denominator for the incidence is the population exposed to the risk of an event. If the event is cardiac surgery and the exposure is type 2 diabetes or smoking, then the population exposed would be all people with diabetes or all smokers. Certainly not all people undergoing cardiac surgery. If the authors consider as the event “a person having diabetes AND undergoing cardiac surgery” then the population at risk would be all people with cardiac disease, with and w/o diabetes.

Even if we make several reasonable assumptions: the need for cardiac surgeries among people without diabetes remains constant; the number of people at risk, with DM or smokers remain constant throughout the change of season, it is unclear why the authors present their results as incidence variations.

For example, if among 100 people undergoing cardiac surgeries in one month there are 30 with diabetes, and the following month the number of people with diabetes who have surgery doubles because of temperature variation, then we would have a proportion of surgeries associated with diabetes, given the above assumptions, from 30% to 46% which is not the measure of incidence variation.”

**Reply:** We thank the Reviewer for appreciating our efforts to revise our paper based on the previous highly pertinent comments. We are also indebted for highlighting the remaining concern with the use of “incidence” as a potentially misleading terminology. We fully accept the detailed explanation of the correct interpretation of our data and changed the term “*incidence*” into “*proportion of surgeries* *associated with diabetes/smoking/elderly*” throughout in the revised manuscript.

**Reviewer #3:**

“Thank you for addressing most of the reviewers' comments. However, the explanation about the effect size still remains unclear. I understand that the percentage of change (variation) is the main effect size for the association between seasonal variation and health parameters. Is that right? Please be more concise and explicit in this regard.”

**Reply:** We are grateful to the Reviewer for acknowledging our revision and we thank for noting for the need of clarifying this methodological detail. In agreement with the Reviewer’s statement, the percentage of change (variation) is the main effect size for the association between seasonal variation and health parameters. This is now specified explicitly in the revised manuscript (page 6, paragraph 1).
